# Medullary radius as a major contributor to variance in the proximal femur: Insights from statistical shape modeling

**DOI:** 10.1111/joa.70064

**Published:** 2025-11-03

**Authors:** Stefan Bracher, Benjamin Haas, Elhadi Sariali, Philippe Zysset

**Affiliations:** ^1^ Musculoskeletal Biomechanics, ARTORG Center for Biomedical Engineering Research, Medical Faculty University of Bern Bern Switzerland; ^2^ Software Engineering and Artificial Intelligence, Symbios Orthopédie SA Yverdon‐les‐Bains Switzerland; ^3^ Service d’orthopédie, hôpital Pitié‐Salpêtrière Paris France

**Keywords:** femoral canal, medullary canal, principal component analysis (PCA), proximal femur, statistical shape model (SSM)

## Abstract

Proximal femoral medullary canal morphology is a key determinant of cementless stem fit, primary stability, and load transfer in total hip arthroplasty (THA), yet population‐level three‐dimensional characterization remains limited. This study was designed to quantify variability in canal geometry and to capture dominant anatomical modes of variation with statistical shape modeling (SSM). Computed tomography data from 763 candidates for primary THA (389 female, 374 male; 20–92 years) were analyzed. Endosteal contours of the proximal canal were processed to build a point‐correspondent SSM by principal component analysis (PCA). Five geometric features were evaluated per specimen: equivalent radius (normalized), roundness, major‐axis angle (torsion), flare index, and curvature. Substantial inter‐individual variability was observed across all features, with differences by sex and age. The first three principal components accounted for 68.4% of total variance, and each showed interpretable associations with at least one geometric feature. Model behavior was examined by synthetic sampling within ±2 SD (specificity) and by 10‐fold cross‐validation (generalization), indicating faithful reconstruction of real shapes and stable performance on held‐out data. These findings provide a compact description of proximal canal shape variation and its key geometric drivers. The resulting population map is expected to support implant selection and sizing in preoperative planning, inform shape‐based classification, and guide design envelopes for standard and personalized stems, with potential efficiencies in manufacturing and material use.

## Graphical abstract

Workflow from CT to PCA‐based shape modeling of the proximal femoral canal (*n* = 763). Feature maps (size, roundness, torsion, flare, curvature) reveal sex‐ and age‐dependent patterns; three PCs capture ≈68% of variance and summarize dominant anatomical changes. Insights inform implant sizing and design envelopes in cementless THA.

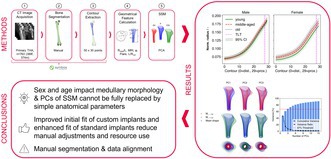

## 1 INTRODUCTION

Femoral morphology has been shown to vary substantially between individuals owing to genetic, lifestyle, and pathological factors (Husmann et al., 1997; Jung et al., 2021; Noble et al., 1988; Veldman et al., 2022). In cementless total hip arthroplasty (THA), the proximal femoral medullary canal is critical because canal geometry influences implant fit, primary stability, and load transfer (Noble et al., 1988; Wettstein et al., 2023). With quantitative computed tomography (QCT), three‐dimensional canal geometry and bone density have been assessed more accurately, and personalized 3D planning has been introduced; however, widely used 2D templating (e.g., the canal flare index (CFI)) captures only part of the geometric complexity (Flecher et al., 2010; Husmann et al., 1997; Noble et al., 1988; Sariali et al., 2012).

For the present analysis, five features were selected – equivalent radius (size), roundness/aspect ratio, major‐axis angle (torsion), curvature, and proximal flare – as a compact description of canal orientation, size, and shape relevant to stem design (Jung et al., 2021; Noble et al., 1988; Zhang et al., 2021). Because these features are interdependent, statistical shape modeling (SSM) with principal component analysis (PCA) was employed to characterize population‐level variation in a low‐dimensional form (Ambellan et al., 2019). Although SSM has been applied to the femur (Boutillon et al., 2022; Chien et al., 2022; Gaffney et al., 2019; La Mattina et al., 2023; Zhang et al., 2016), explicit analyses of the proximal medullary canal remain limited, particularly with sex‐ and age‐stratified cohorts (Ramesh et al., 2025; Zhang et al., 2021).

The clinical and engineering relevance of a population‐based description of proximal canal shape is considerable: such information supports implant selection and sizing, mapping of shape distributions and outliers, development of classification schemes, and definition of design envelopes for standard and patient‐specific stems, with implications for manufacturing efficiency. Despite these potential benefits, comprehensive three‐dimensional characterization of the proximal canal in large primary THA cohorts has been scarce.

Accordingly, the present study was undertaken to quantify variability in major‐axis angle (torsion), equivalent radius, roundness, flare index, and curvature of the proximal femoral canal, and to relate these features to anatomical modes of variation derived from SSM/PCA in a large primary THA cohort, with analyses stratified by sex and age.

## 2 METHODS

### 2.1 Data collection and segmentation

The Cantonal Ethics Committee of Bern determined that the project does not fall within the scope of the Swiss Human Research Act (HRA) and therefore does not require ethics committee approval (Req‐2025‐00130). The committee is not responsible for evaluating the project. Data protection was ensured, and all data were de‐identified prior to analysis. The study was conducted in accordance with the Declaration of Helsinki (Goodyear et al., 2007).

Preoperative CT data of the proximal femur were available for 763 patients who subsequently underwent primary THA (389 female, 374 male; 20–92 years). For analysis, age was grouped into tertiles: 20–52 years (*n* = 256), 53–65 years (*n* = 263), and ≥66 years (*n* = 244). Information on lifestyle, medication, and comorbidities was unavailable.

Imaging followed the HIP PLAN® protocol (Symbios Orthopédie SA, Yverdon‐les‐Bains, Switzerland), which includes hip, knee, and ankle scans and enables calculation of full femur and leg length (Sariali et al., 2009). Segmentation, landmark annotation, and femur‐length measurement were performed by Symbios using internal software. Segmentation used a slice‐wise, semi‐automatic, spline‐based workflow: on each axial section (typically 15–30 slices per case with 5–10 mm spacing), splines were initialized to approximate the external cortex and the endosteal surface (50 control points per slice), with additional spline steps around the greater trochanter/femoral head to capture local morphology; operators then manually refined contours on every slice. Scans were not required to be metal‐free; when ipsi‐ or contralateral implants were present, artifacts could increase the amount of manual editing, and operators selected an alternative preset to improve delineation near metal. To ensure consistency, the task was performed by trained staff, and all cases underwent independent second‐person review prior to finalization. Landmarks were defined manually; the most superior points on the greater and lesser trochanter were designated GT and TLT, respectively, and femur length was measured from GT to the intercondylar fossa. The authors received the proximal endosteal contour data (15–30 axial slices per case, 5–10 mm spacing, 50 points per slice), together with calculated femur lengths and demographic information.

### 2.2 Preprocessing

Contour data were imported into Python. Inter‐slice spacing was reduced by linear interpolation between adjacent sections to create 100 slices prior to trimming. Canals were then trimmed proximally at 10% of total femur length distal to the GT and distally so that the retained segment equaled 22.1% of femur length; this was chosen to maximize the overlap of a comparable anatomical region across cases. The resulting segments measured 94.2 ± 7.7 mm in length (65.1–123.6 mm). Left femora were mirrored about the sagittal plane (*x*‐axis) so that all specimens were represented as right‐sided.

Contours were translated so that the centroid lay at the origin. A minimum‐volume enclosing ellipsoid was then fitted (Moshtagh, 2005), and each specimen was rotated to align the Cartesian axes with the ellipsoid's principal axes, thereby standardizing orientation across femora. Alignment to the anatomical/mechanical axis was not feasible because distal condyles were unavailable; the ellipsoid‐based alignment provided a consistent proxy. The *x* and *y* axes were treated as pseudo mediolateral (ML) and anteroposterior (AP) directions, respectively.

**FIGURE 1:**
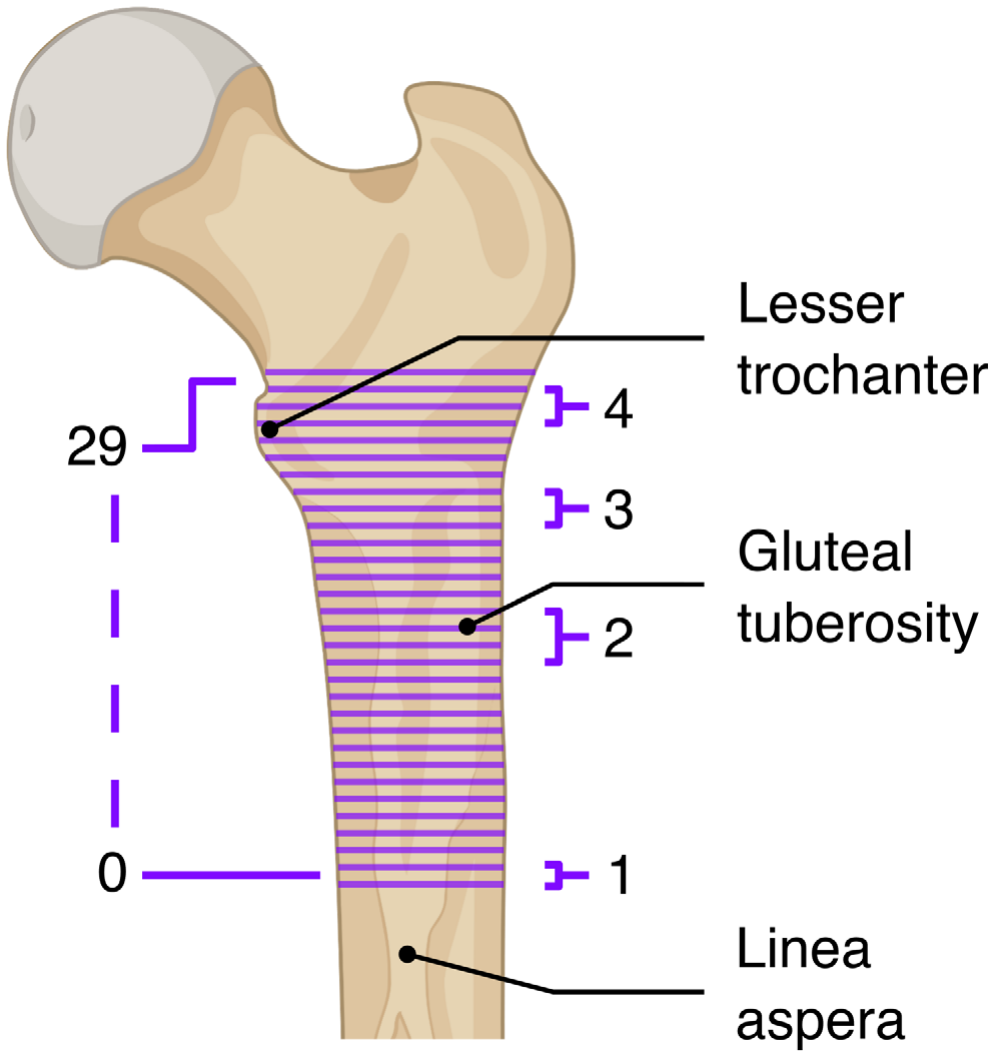
Anatomical reference of proximal femur. Posterior view of the right proximal femur showing the 30 contour levels (labeled 0–29) used for shape analysis after resampling. The proximal canal was subdivided into four regions of interest to indicate locations where the evaluated geometric parameters showed notable changes. Anatomical features are also indicated. Created in BioRender. Bracher, S. (2025) https://BioRender.com/r48d486

Because alignment moved sections off the original CT planes, cross‐sections were resliced in the aligned coordinate system (linear interpolation) to obtain 30 uniformly spaced planar sections, labeled 0–29 from distal to proximal (Figure 1). Point‐to‐point correspondence was established per section by fitting a closed cubic spline to the endosteal perimeter, defining a consistent start at the intersection with the posterior ray through the section centroid, and sampling 50 points at equal arc‐length intervals.

### 2.3 Calculation of geometrical features

For each contour, the equivalent radius *r*_*eq*_ was defined as $r_{\mathrm{eq}}=A/\pi$, where *A* was the contour area obtained from the ordered perimeter points (polygonal approximation). A normalized radius was then computed as *r*_*eq*_ */L*, with *L* denoting the canal segment length. Roundness was quantified as the medullary roundness index (MRI), the ratio of ellipse major‐to‐minor axes fitted to the contour points (Halíř & Flusser, 1998). The major‐axis angle (*φ*) was defined as the angle between the ellipse's major axis and the *x*‐axis and was used as a per‐contour measure of torsion. The flare index was defined at the specimen level as the ratio of the largest to the smallest equivalent radius observed across the 30 sections, capturing the degree of proximal flare. Curvature was obtained as the reciprocal of the radius of a least‐squares sphere fitted to the 3D sequence of section centroids, and a dimensionless normalized curvature was reported as the segment length divided by that radius.

To relate SSM scores to geometry, a single specimen‐level summary was computed for each feature: mean normalized radius, mean *MRI*, overall torsion (*Δφ* between distal contour 0 and proximal contour 29), flare index (max/min *r*_*eq*_ across the 30 contours), and normalized curvature. Shapes were isotropically scaled so that the longitudinal (*z*) extent spanned 0–1. All feature computations were performed per specimen prior to PCA and independently of the SSM; the ±2 SD renderings were used only for qualitative visualization.

### 2.4 SSM

The generation of SSM models of the medullary canal depended upon PCA on point‐correspondent shapes (1500 points/shape) in Python (PySSAM) (Williams et al., 2023). PCs (eigenvectors (*υ*_*i*_) with eigenvalues (*λ*_*i*_)) were obtained from the covariance matrix, and PC scores were obtained by summing the products of each PC's eigenvectors and the individual standardized coordinates of the contour points. PCA is explained in more detail elsewhere (Jolliffe, 2002). Shape variation was visualized as

$$X_{i,\pm 2\sigma }=\bar{\mu }\pm \upsilon_{i}\cdot 2\sqrt{\lambda_{i}}$$

where $X_{i,\pm 2\sigma }$ corresponds to the two geometries obtained by altering the mean shape ($\bar{\mu }$) by ±2 standard deviations (SD). In addition to the pooled model (*n* = 763), sex‐specific SSMs (female *n* = 389; male *n* = 374) were built with the identical pipeline for descriptive comparison only. Compactness was assessed from the variance spectrum (per‐PC variance and cumulative variance), and three PCs were retained using a scree/elbow criterion. Specificity was evaluated by sampling synthetic shapes within ±2 SD along PCs 1–3, reconstructing them in the standardized correspondence space, and computing each sample's nearest‐neighbor Euclidean distance to the training set; the distribution was compared to real‐to‐real nearest‐neighbor distances. Generalization was tested by 10‐fold cross‐validation: in each fold, a model was fit on 90% of shapes, the remaining 10% were projected onto the retained PCs, and reconstruction error was recorded as scaled MSE (unitless) (Styner et al., 2003).

### 2.5 Statistical analysis

Continuous variables were tested for normality with the Shapiro–Wilk test. Between‐group comparisons used independent samples t‐tests (normal data) or Mann–Whitney U‐tests (non‐normal). The effects of sex and age group on mean *r*_*eq*_ */L*, mean MRI, and mean *φ* were analyzed with two‐way ANOVA (sex × age) for each contour, with Tukey's HSD for post‐hoc pairwise comparisons. Associations between per‐specimen PC scores and geometric features were assessed by simple linear regression (ordinary least squares); results are summarized by *R* ^*2*^ and two‐sided *p* values. Unless stated otherwise, data are reported as mean ± SD, and statistical significance was set at *α* = 0.05 (two‐sided). Analyses were performed in Python (Statsmodels, SciPy) (Van Rossum & Drake, 2009).

## 3 RESULTS

### 3.1 Geometrical features of cohort

**FIGURE 2:**
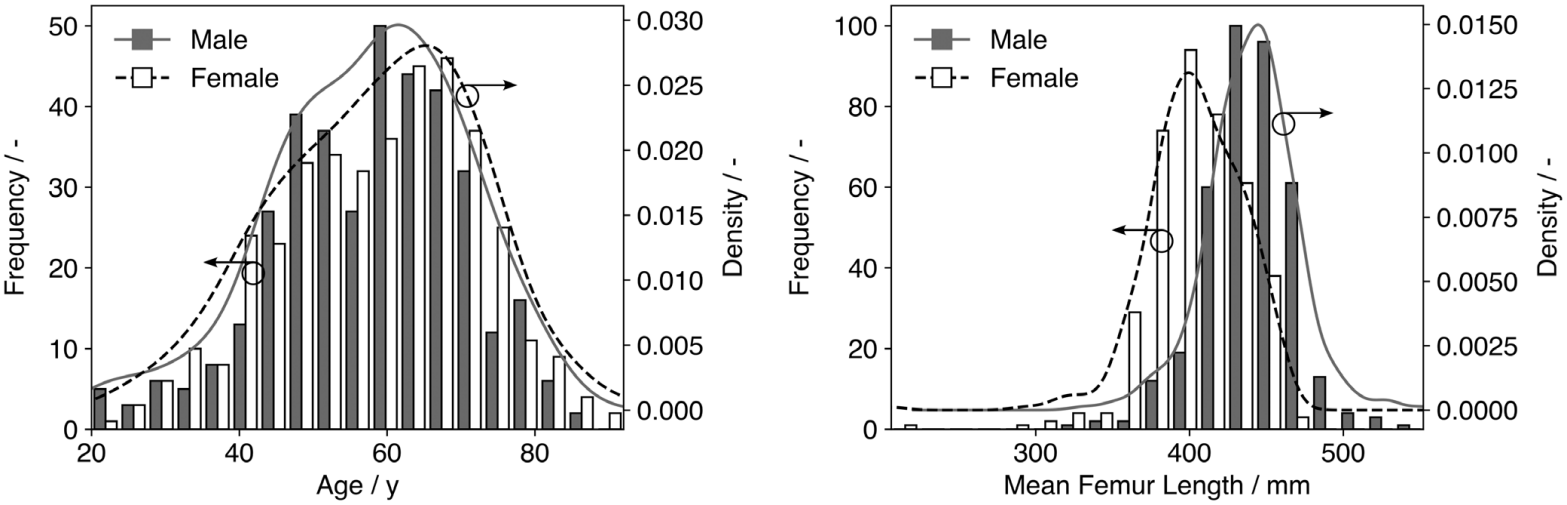
Cohort characteristics by sex. Histograms with kernel density estimates showing age (left) and mean femur length (right) for female (white) and male (gray) cohorts. Group summary statistics and corresponding *p* values are reported in the text.

Age distributions did not differ by sex (female: 59 ± 13 years, 22–92; male: 58 ± 13 years, 20–87; *p* = 0.33). Femur length was greater in males than in females (male: 440 ± 29 mm, 331–552; female: 400 ± 31 mm, 206–466; *p* < 0.001). Age and femur‐length histograms by sex are shown in Figure 2.

**FIGURE 3:**
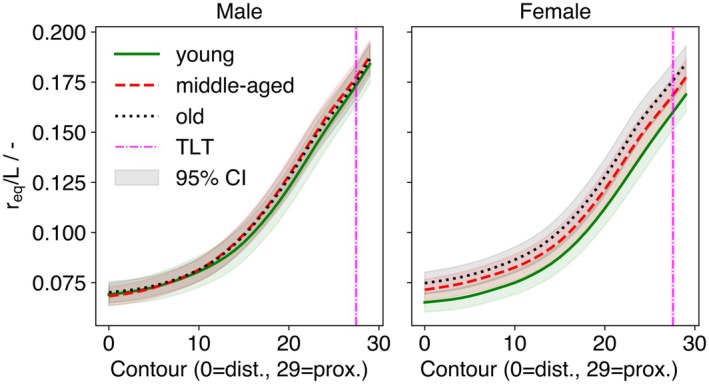
Variability in canal radius by sex and age. Mean normalized equivalent radius (*r*_*eq*_ */L*) across contours with 95% CI shading; The vertical magenta line marks the TLT level.

Mean normalized equivalent radius (*r*_*eq*_ */L*) by contour (0–29) is shown separately by sex (Figure 3). In females, *r*_*eq*_ */L* varied with age and exhibited a flatter proximal increase in older groups: significant differences were observed for young vs. middle‐aged (*p* < 0.01) and young vs. old (*p* < 0.001) at all contours, and for middle‐aged vs. old at contours 24–29 (*p* < 0.05). In males, no significant age effect was detected (Figure S1).

**FIGURE 4:**
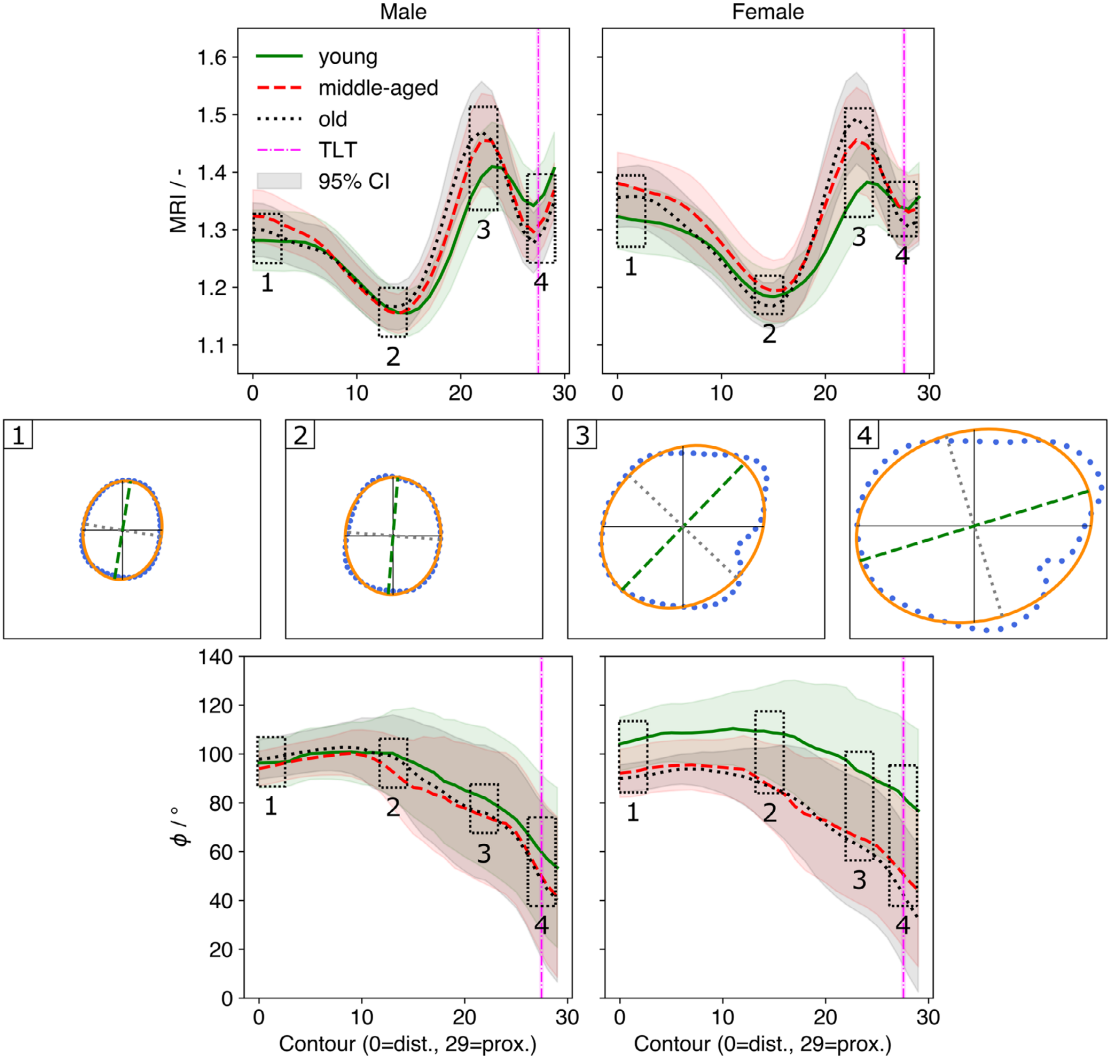
Medullary roundness (MRI) and major‐axis angle (φ) by sex, age, and contour. Top: Mean MRI across contours with 95% CI shading; line style denotes age group. The vertical magenta line marks the TLT level. Middle: Representative superior‐view cross‐sections at numbered ROIs 1–4 (as in Figure 1), showing raw endosteal points (blue), ellipse fit (orange), major axis (green dashed), and minor axis (gray dotted). Bottom: Mean major‐axis angle φ with 95% CI shading across contours; dashed boxes indicate the locations corresponding to ROIs 1–4 above.

Mean *MRI* (±95% CI) by contour and age group is shown separately for males and females (Figure 4). Sex differences in *MRI* were present at contours 0–15, 19–21, and 25–29 (*p* < 0.05). Age effects within the male cohort were detected for young vs. middle‐aged at contours 0–1, 19–21, and 27, and for young vs. old at 16–22 and 26–29. In females, age effects were observed for young vs. middle‐aged at 0–6 and 19–23 (*p* < 0.03) and for young vs. old at 19–24 (*p* < 0.02) (Figure S1). Across sexes, the *MRI* profile exhibited two local minima at contours 12–15 and 27–29, with a peak at 22–25.

No overall sex difference in *φ* was detected, but results were not pooled because of age effects (Figure 4). In males, age had no significant effect on *φ* (*p* > 0.05; Figure S1), although the younger group tended to show higher values at contours 12–29. In females, age effects were significant at all contours, with larger angles in the young vs. middle‐aged and young vs. old comparisons (*p* < 0.001). The contour‐wise pattern was similar between sexes, but males exhibited a steeper proximal decline around contours 12–15 and 27–29, mirroring the *MRI* pattern (Figure 4). Confidence intervals were relatively wide, indicating substantial inter‐individual variability.

**FIGURE 5:**
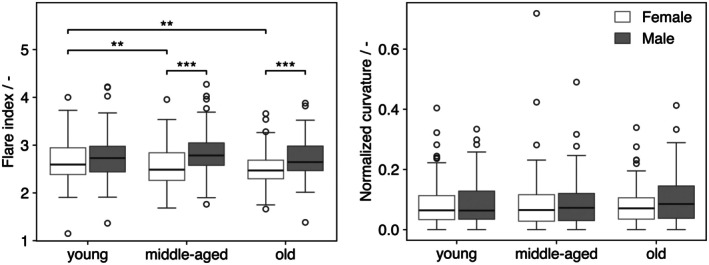
Boxplots of flare index (max/min r_eq_ across 30 contours) and normalized curvature (*L/R*), stratified by sex (white = female, gray = male) and age group. Brackets indicate Tukey HSD post‐hoc comparisons; significance codes: *p < 0.05, **p < 0.01, ***p < 0.001.

A sex effect was detected for the flare index (males higher; *p* < 0.001), whereas the main effect of age was not significant (*p* > 0.05). Post‐hoc comparisons showed that males had greater flare indices than females within the middle‐aged and old groups (*p* < 0.001). Within sex, no age differences were observed in males; in females, the young group exhibited higher values than both middle‐aged (*p* < 0.03) and old (*p* < 0.003) groups, indicating an age‐related decrease confined to females (Figure 5).

Normalized curvature showed no significant effects of sex or age, although a nonsignificant tendency toward higher values in the old group – more apparent in males – was noted (Figure 5).

### 3.2 SSM

**FIGURE 6:**
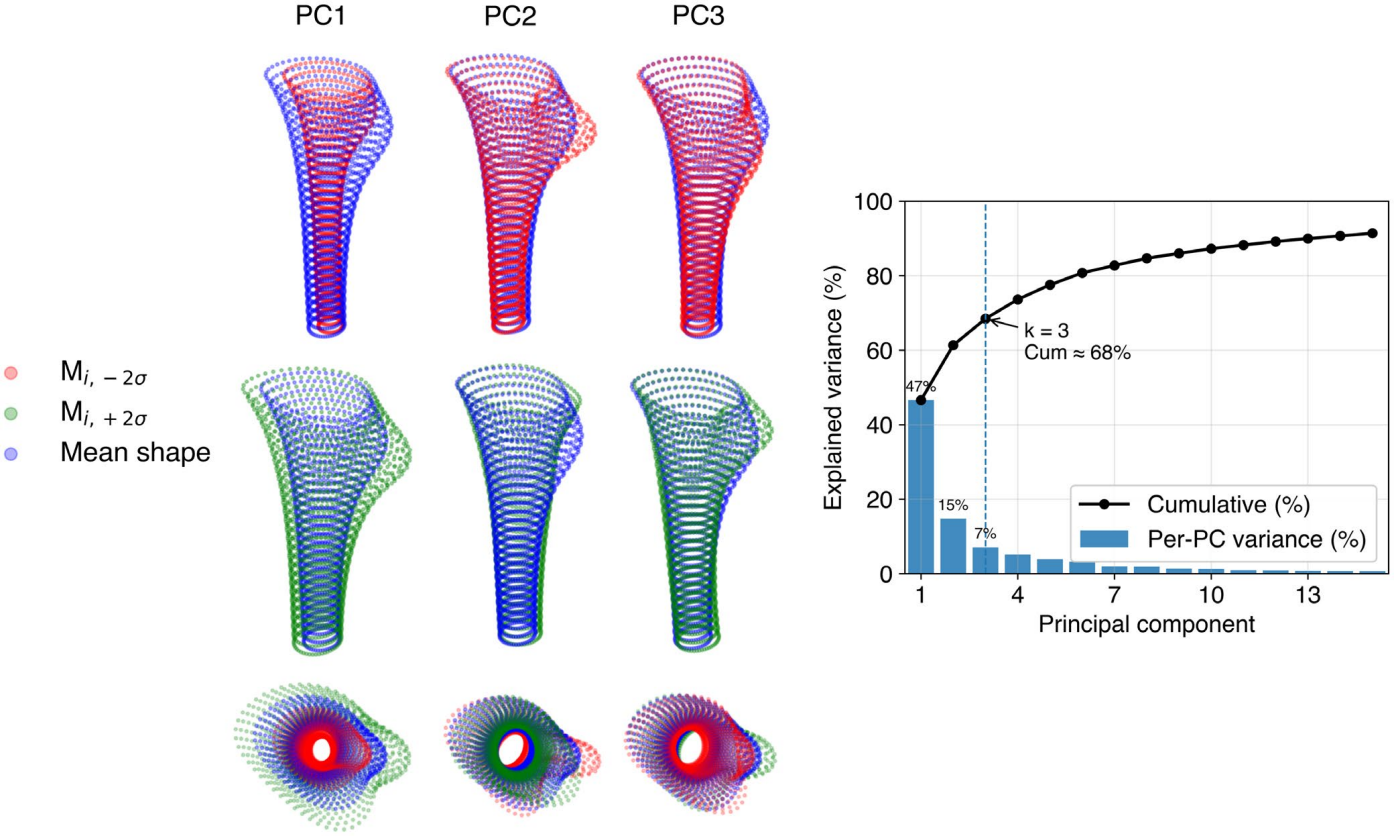
PC variation in canal shape (only used for illustrative purposes). Anterior and superior views of mean medullary canal (blue) and deformations along the first three PCs at ±2 SD. Right: Scree plot showing per‐component explained variance (bars) and cumulative explained variance (line) for the first 15 PCs; the scree/elbow selection at PC = 3 is indicated (cumulative ≈68%).

Three modes or PCs were required to explain approximately 68.4% of the variance in the population, while they accounted for 46.6%, 14.7%, and 7.1%, respectively (Figure 6). Despite this reduction, training‐set reconstructions with three PCs showed very low errors, indicating that the dominant variation is retained (Figure S2). Shapes sampled from PCs 1–3 within ±2 SD showed nearest‐neighbor distances to the real set that overlapped the real/real baseline (synthetic/real: 0.482 ± 0.083, median 0.474; real/real: 0.638 ± 0.254, median 0.586; unitless, standardized space) (Figure S3). 0% of synthetic samples exceeded the real 95th percentile (1.041), indicating high specificity. In 10‐fold cross‐validation, reconstruction errors on held‐out shapes were indistinguishable from training errors (scaled MSE: train 5.752 × 10^−1^ ± 1.33 × 10^−3^ vs. test 5.752 × 10^−1^ ± 1.33 × 10^−3^; medians 5.753 × 10^−1^ and 5.752 × 10^−1^, respectively) (Figure S4). The ±2 SD renderings in Figure 6 are illustrative of deformation patterns and were not used for statistical inference.

The first three modes of the pooled SSM are shown in Figure 6 (mean shape and ± 2 SD deformations). Associations between PC scores and geometric features are quantified in Table 1 and Figure S3: PC1 showed the strongest relationship with normalized radius (*r*_*eq*_ */L*) and a weaker relationship with curvature; PC2 correlated with roundness, overall torsion (*Δφ*), and the flare index, and, on visual inspection, also modulated the expression of the lesser trochanter; PC3 related primarily to the flare index. Apart from the PC1‐*r*_*eq*_ */L* association, the correlations were predominantly small. Sex‐specific SSMs yielded qualitatively similar mean shapes and modes; the variance explained by PCs 1–3 was 50.0%, 13.4%, and 7.2% in females and 43.6%, 16.6%, and 7.3% in males (Figure S5).

**TABLE 1:** Correlations between principal components and geometric features (pooled cohort, *n* = 763).

Characteristic	Statistics	PC1 (−)	PC2 (−)	PC3 (−)
Mean normalized radius	*R* ^*2*^	**0.95**	0.04	0.01
	*P*	**<0.001**	<0.001	<0.001
	95% CI	**[48.863, 50.486]**	[6.006, 13.131]	[1.852, 9.064]
Mean roundness	*R* ^*2*^	0.01	**0.16**	0.01
	*P*	<0.05	**<0.001**	<0.001
	95% CI	[0.016, 0.825]	**[−2.670, −1.927]**	[0.271, 1.077]
Overall torsion	*R* ^*2*^	0.05	**0.08**	0.03
	*P*	<0.001	**<0.001**	<0.001
	95% CI	[0.005, 0.009]	**[−0.011, −0.006]**	[0.003, 0.008]
Flare index	*R* ^*2*^	0.03	**0.11**	**0.11**
	*P*	<0.001	**<0.001**	**<0.001**
	95% CI	[−0.593, −0.270]	**[−0.905, −0.595]**	**[−0.915, −0.605]**
Normalized curvature	*R* ^*2*^	**0.03**	0.00	0.00
	*P*	**<0.001**	>0.05	>0.05
	95% CI	**[0.614, 1.495]**	[−0.250, 0.643]	[−0.826, 0.066]

*Note*: Linear regressions of PC scores against per‐specimen features are reported as *R* ^*2*^, *P* value, and 95% CI of the slope. Bold highlights the strongest significant associations. Scatterplots are provided in Figure S6.

**FIGURE 7:**
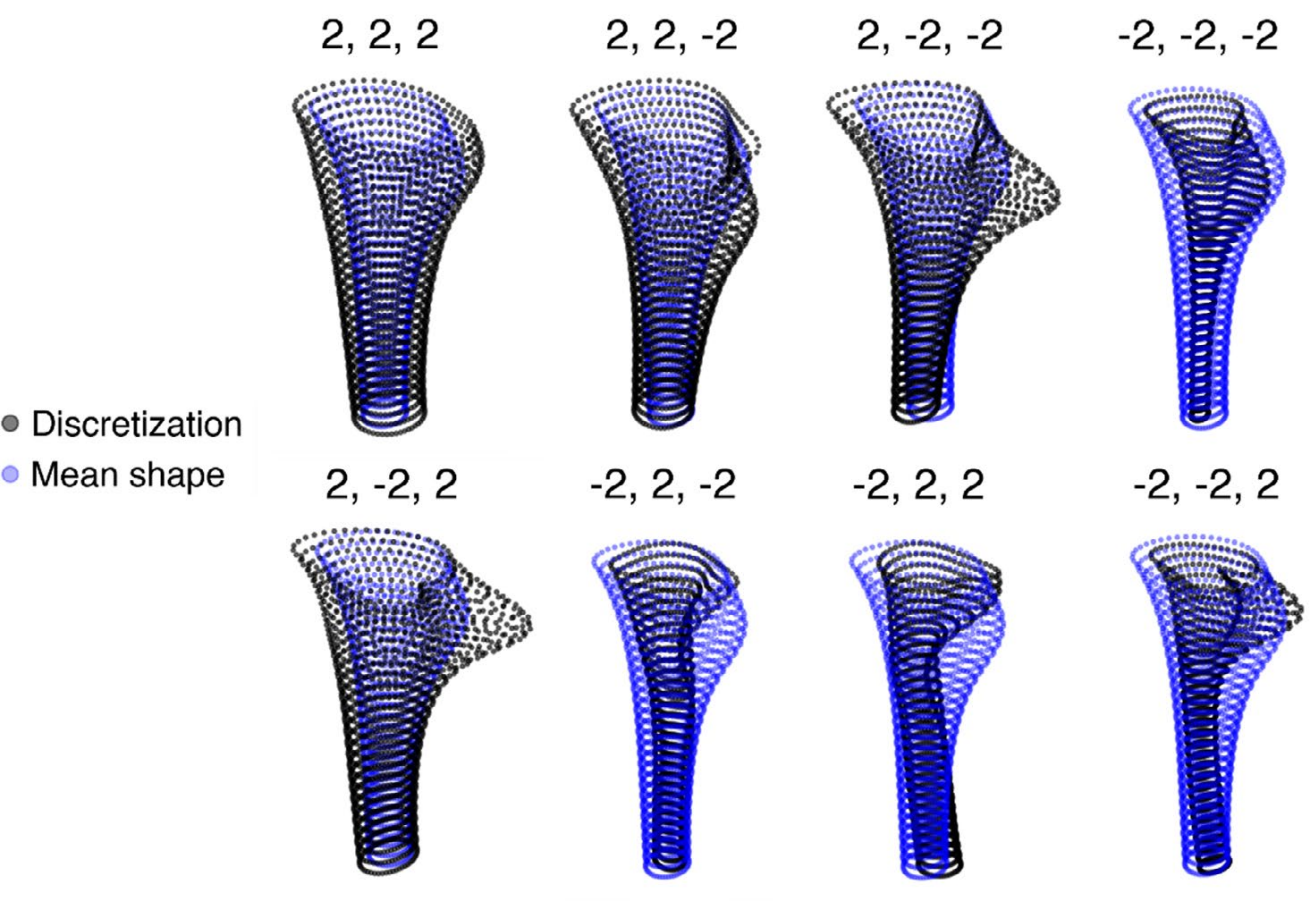
Synthesized canal shapes from principal component combinations. Anterior views of shapes generated by adding ±2 SD along PC1‐PC3 from the mean; the triplet above each panel gives the coefficients in the order (PC1, PC2, PC3). The generated shape is shown as black points (discretization), overlaid on the mean shape in blue. Examples illustrate the range and interactions captured by the first three PCs; they were used for visualization only.

Pairwise score plots of the first three PCs showed no distinct clustering or separation (Figure S7), indicating a largely continuous distribution of canal shapes rather than discrete subgroups. The same pattern was observed when scores were examined within sex and age strata (data not shown). Figure 7 visualizes joint variation by sampling the corners of the 3‐D score space for PCs 1–3 (all ±2 SD combinations), illustrating how modes co‐occur in the cohort; these panels are illustrative only and were not used for statistical inference. Once more, the influence of the first PC is visible through a change in the aspect ratio. Additionally, alterations in the expression of the lesser trochanter, curvature, roundness, overall torsion, and flare index, introduced by PC2 and PC3, are evident.

## 4 DISCUSSION

A statistical shape‐modeling framework was developed from proximal femoral canal contours of 763 primary THA patients (20–92 years) to quantify geometric features and examine how they relate to anatomical variation.

### 4.1 Geometrical features of cohort

Five canal features were analyzed – normalized equivalent radius (*r*_*eq*_ */L*), *MRI*, major‐axis angle (*φ*), flare index, and normalized curvature – stratified by sex and age (young, middle‐aged, old).

A clear sex effect was observed for *r*_*eq*_ */L*, with higher values in males and a progressive proximal increase in both sexes (Figure 3). In females, *r*_*eq*_ */L* increased with age – significant for young vs. middle‐aged and young vs. old at all contours, and for middle‐aged vs. old at proximal contours – whereas males showed no age effect (Figure S1). These findings are consistent with prior work on canal size (Laine et al., 2000; Noble et al., 1988; Sen et al., 2010; Zhang et al., 2021) and align with age‐related endosteal expansion driven by endosteal resorption and periosteal apposition, accentuated after menopause (Compston et al., 1989; Parfitt, 1984; Roberts et al., 2016).

The *MRI* was significantly higher in females than in males at several contours and followed a consistent profile in both sexes, with two minima at contours 12–15 and 27–29 and a peak at 22–25 (Figure 4). The rise in *MRI* from ROI 2 to ROI 3 is plausibly explained by the gluteal tuberosity, which widens the canal asymmetrically. Approaching the lesser trochanter, *MRI* then declines toward ROI 4, indicating a more circular proximal cross‐section. Comparable magnitudes have been reported by Zhang et al. for a related roundness metric – defined as the ratio of the canal's longest diameter to the perpendicular diameter through its midpoint – though that study did not stratify by sex or age (Zhang et al., 2021). With respect to age, the *MRI* profile in younger individuals was shifted, showing a lower peak at 22–25 but a higher proximal minimum at 27–29 in both sexes; the difference was reduced yet still present when middle‐aged and older groups were compared (Figure S1). These patterns are consistent with age‐related changes in bone microarchitecture driven by altered musculoskeletal loading, with site‐specific remodeling (endosteal resorption and periosteal apposition) producing differential changes in canal eccentricity across the cross‐section (Compston et al., 1989; Parfitt, 1984; Roberts et al., 2016).

The major‐axis angle (*φ*) was used as a contour‐wise proxy for canal torsion. In line with our results, age‐related increases in *φ* were confined to women (middle‐aged and old > young), whereas no age effect was detected in men, consistent with (Zhang et al., 2021). A plausible explanation is menopause‐related endosteal resorption with compensatory periosteal apposition, together with age‐related shifts in habitual loading, which can alter cross‐sectional eccentricity and its orientation and thereby increase apparent twist along the proximal canal. It should be noted that *φ* reflects the orientation of an ellipse fitted to each cross‐section rather than centerline torsion per se, but it provides a stable, anatomically interpretable surrogate across the resliced sections.

The flare index was sex dependent, with higher values in males – particularly in the middle‐aged and old groups – while no overall main effect of age was detected. Within‐sex contrasts indicated an age‐related decline in females but not in males. Higher male values may be compatible with greater cortical shell thickness and proximal flare reported elsewhere (Casper et al., 2012), though the cortex was not measured here. Direct numerical comparison with the traditional 2D CFI is not appropriate because the present metric reflects 3D endosteal geometry, whereas CFI is derived from AP radiographs (Noble et al., 1988). The observed tendency toward a more cylindrical canal with age may reflect age‐related alterations in gait mechanics – reduced ankle push‐off with greater hip contribution – and task‐dependent increases in frontal‐plane stability demands, which together plausibly modify the proximal canal's loading environment (Boyer et al., 2023; DeVita & Hortobagyi, 2000; Hurt & Grabiner, 2015; Lewis & Ferris, 2008).

Normalized curvature showed no significant effects of sex or age; only a small, nonsignificant tendency toward higher values in the old group – more apparent in males – was observed (Figure 5). This contrasts with reports linking femoral curvature to older age, sex, race, shorter stature, and lower BMD (with females often exhibiting greater curvature) (Furihata et al., 2021; Lu et al., 2012; Shimosawa et al., 2019). A likely explanation is methodological: curvature here was quantified over a short proximal segment that is anatomically relatively straight, so age/sex effects seen in more mid–distal diaphyseal regions may be attenuated. In terms of absolute magnitude, our curvature values are consistent with prior work (Betti et al., 2024; Harma et al., 2005; Lu et al., 2012; Shimosawa et al., 2019).

### 4.2 SSM

The first three PCs captured 68% of total variance (PC1 46.6%, PC2 14.7%, PC3 7.1%). Regression of PC scores on geometric features (Table 1; Figure S3), together with ±2 SD deformations (Figure 6), indicated that PC1 primarily modulated normalized radius (*r*_*eq*_ */L*) with a weaker association to curvature; PC2 reflected changes in roundness, overall torsion (*Δφ*), and the flare index, and – on visual inspection – altered the expression of the lesser trochanter; PC3 related chiefly to the flare index. These observations are consistent with prior work in which a radius‐equivalent measure aligned with PC1 (Ramesh et al., 2025). Differences in higher modes likely reflect the larger proximal extent modeled in that study, where PC2–PC5 influenced calcar rotation, femoral version, varus/valgus, and shaft torsion.

Sex‐specific SSMs yielded qualitatively similar mean shapes and modes. As expected, a global size mode was suppressed because shapes were normalized along the longitudinal axis before PCA; by contrast, studies that did not normalize reported size‐dominated PC1 (Betti et al., 2024). Pairwise score plots of PC1–PC3 showed no clustering (Figure S7), and the same continuous distribution was observed when stratified by sex and age, suggesting that discrete subtypes are not captured by the first three modes.

Apart from the strong PC1‐*r*_*eq*_ */L* relationship, feature‐PC correlations were modest (Table 1), indicating that the PCs encode composite shape changes not fully summarized by single scalar descriptors. Nevertheless, sampling combinations of the first three PCs (±2 SD; Figure 7) provided a compact way to explore the principal shape spectrum of the proximal canal. In design contexts, such synthesized shapes can support pre‐shaping toward patient anatomy and reduce manual adjustments, with potential downstream benefits for manufacturing efficiency and material use.

### 4.3 Limitations

The cohort comprised patients planned for personalized cementless stems, which likely enriched atypical morphologies. While this “stress‐tests” the design space, generalizability to asymptomatic populations or standard‐implant candidates is limited; inclusion of such comparators in future work would strengthen external validity.

Only proximal endosteal contours and derived demographics were provided. Consequently, whole‐femur references (e.g., femoral neck axis, bicondylar plane) were unavailable, and alignment to anatomical/mechanical axes could not be performed. Segmentation and landmarking were conducted by an external provider using proprietary parameters; threshold values and potential acquisition/segmentation variability (e.g., metal artifacts, operator effects) could not be audited.

Alignment was found to influence the distribution of variance across early PCs. Landmark‐based schemes were tested but were sensitive to operator variability; a landmark‐independent, ellipsoid‐based alignment with reslicing was therefore adopted. Although qualitative shape trends were stable, alternative alignment choices can reorder modes, as reported by (Moraiti et al., 2024). This should be considered when comparing SSMs across studies.

### 4.4 Conclusion

A large, CT‐based cohort (*n* = 763) was analyzed to quantify proximal medullary canal geometry (normalized radius, roundness, major‐axis angle as a proxy for torsion, flare index, curvature) by sex and age, and to model shape variation using SSM. Substantial inter‐individual variability was observed. Normalized radius was higher in males and increased with age in females, while torsion showed age‐related increases confined to females; flare was greater in males with an age‐related decline in females; curvature showed no significant sex‐ or age‐related effects over the short proximal segment examined. An SSM retaining three principal components (≈68% variance) captured the dominant modes of variation: PC1 aligned chiefly with normalized radius, PC2 with roundness, torsion, and flare, and PC3 with flare. Aside from the strong PC–radius relationship, feature‐PC correlations were modest, indicating that the leading modes encode composite shape changes that cannot be reduced to single scalar descriptors. Representative canals synthesized from combinations of the first three PCs illustrated the principal shape spectrum and offer a compact path to pre‐shaping toward patient anatomy. Taken together, these results provide a data‐driven description of proximal canal diversity and a low‐dimensional model that could aid patient‐specific stem design and selection by reducing manual adjustments and streamlining manufacturing, while inviting future validation in external cohorts and with whole‐femur anatomical references.

## AUTHOR CONTRIBUTIONS

**Stefan Bracher:** Methodology, Software, Validation, Formal analysis, Data Curation, Writing – Original Draft, Writing – Review and Editing, Visualization. **Benjamin Haas**: Resources, Data Curation, Writing – Review and Editing. **Elhadi Sariali**: Writing – Review and Editing. **Philippe Zysset:** Conceptualization, Methodology, Validation, Resources, Supervision, Project administration, Funding acquisition, Writing – Review and Editing.

## Supporting information


Data S1:

## Data Availability

Research data are not shared.
