# Supplementary material for: Medullary radius as a major contributor to variance in the proximal femur: Insights from statistical shape modeling
Source: J Anat. 2025 Nov 3;248(5):766–76. doi: 10.1111/joa.70064 (PMC13069162; doi:10.1111/joa.70064)
Supplement: Supplementary file 1 — Data S1: [file JOA-248-766-s001.docx]

**Supplementary Material**

**Contour and sex specific p-values of geometric parameters**


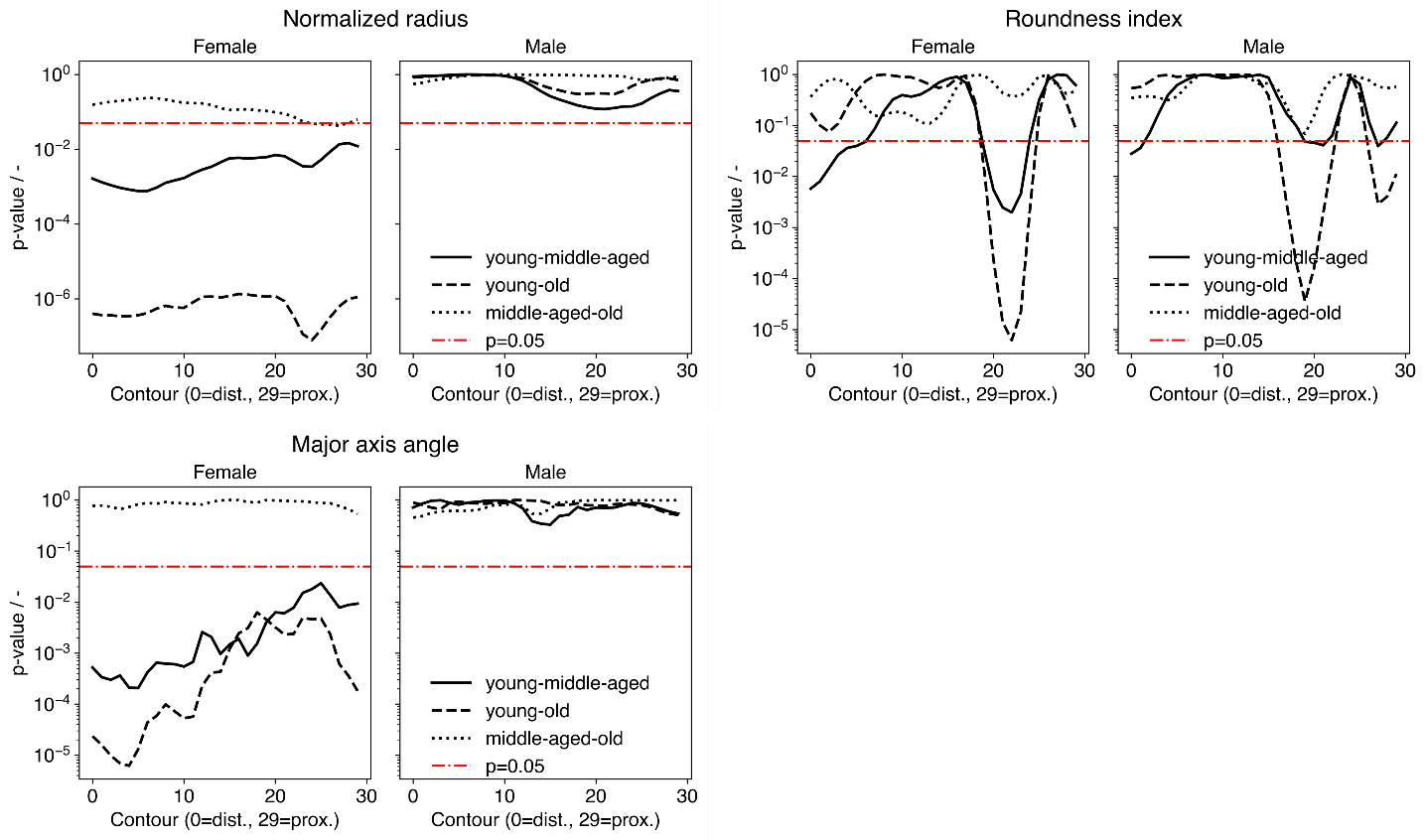


Fig. S-1 Contour and sex-specific p-values for normalized radius, roundness index, and major axis angle.

**Evaluation of statistical shape model**

**Notation and setup**

Each femoral canal shape is represented by $P=1500$ 3D points in fixed correspondence (30 contours × 50 points). After preprocessing/alignment and isotropic scaling (standardizing longitudinal extent), each shape is vectorized as $x_{i}\in\mathbb{R}^{3p}, i=1,\ldots,N$. Let $\mu$ be the sample mean and $C$ the covariance of $\{x_{i}\}$. PCA of $C$ yields eigenpairs $(\lambda_{j}, v_{j})$ with $\lambda_{1}\geq\lambda_{2}\geq\ldots$, and $V_{k}=\left[ v_{1},\ldots, v_{k} \right]\in\mathbb{R}^{3p\times k}$.

In PCA, each $\lambda_{j}$ is the variance (“spread”) along mode $v_{j}$; larger $\lambda_{j}$ indicates a more important anatomical variation. Distributions of per-shape reconstruction errors with $k=3$ PCs are shown in Fig. S-2 (scaled and mm²). Scree plots for sex-specific models are depicted in Fig. S- 5.


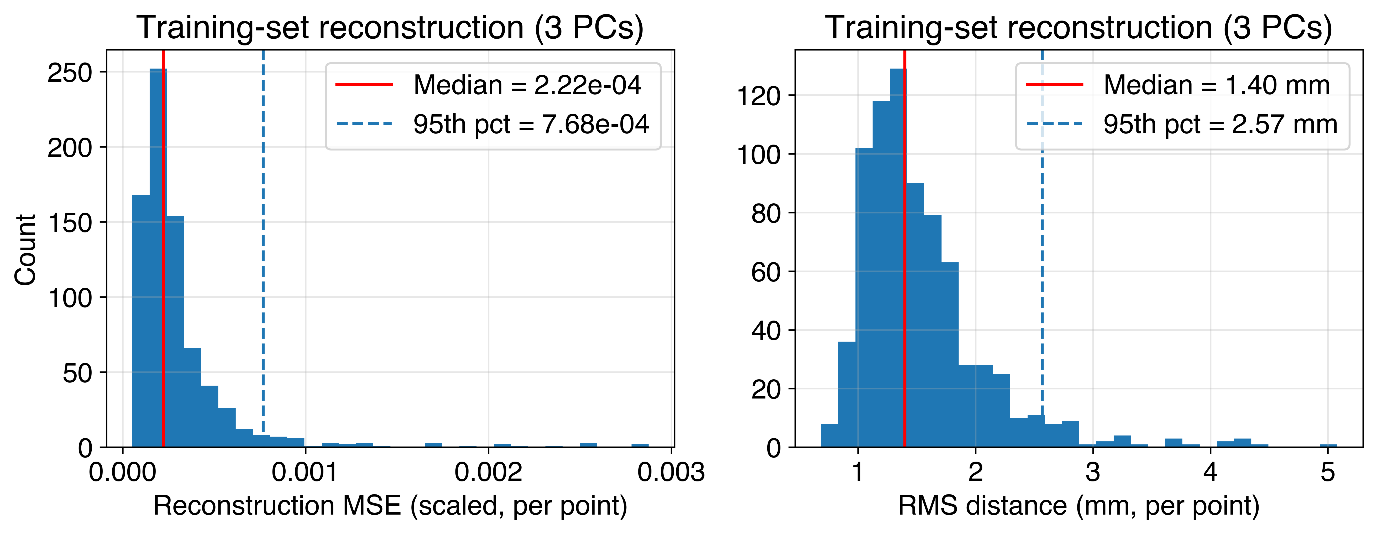


Fig. S-2 Per point training-set reconstruction error with three PCs. Histograms of per-shape reconstruction error for the pooled SSM (n = 763). Left: MSE in the standardized (unitless) correspondence space. Right: RMS in physical units (mm). Solid red line = median; dashed blue line = 95th percentile.

**Compactness (how efficiently few modes summarize variability)**

$$Compactness\left( k \right)=\frac{\sum_{j=1}^{k} \lambda_{j}}{\sum_{j=1}^{3P} \lambda_{j}}$$

Describes the fraction of total shape variability captured by the first k PCs; the “elbow” indicates diminishing returns (Fig. 6). Sex-specific scree curves are provided in Fig. S- 5.

**Specificity (do sampled shapes look like real anatomy?)**

With $k$ PCs, draw scores

$$s\sim\mathcal{N}\left( 0, diag\left( \lambda_{1},\ldots,\lambda_{k} \right) \right),$$

where $\mathcal{N}$ corresponds to a normal (Gaussian) distribution. Then truncate each component to ±2 SD (main analysis), and synthesize

$$\tilde{x}=\mu+V_{k}s.$$

Using Euclidean distance in correspondence space the ℓ^2^ distance between vectorized shapes after alignment and isotropic scaling for shapes *a*, *b* with P corresponding 3D points *a_p_*, *b_p_*,

$$d\left( a,b \right)=\left\| a-b \right\|_{2}=\left( \sum_{p=1}^{P} \left\| a_{p}-b_{p} \right\|_{2}^{2} \right)^{1/2},$$

compute for each synthetic $\tilde{x}$ its nearest-neighbor (NN) distance to the real training set, ${min}_{j} d(\tilde{x},x_{j})$. As a baseline of natural variability, compute “real 🡪 real” NN distances ${min}_{j\neq i} d(x_{i},x_{j})$.

If synthetic 🡪 real distances overlap the real 🡪 real baseline (similar mean/median) and rarely exceed its 95th percentile, the model samples anatomically plausible shapes. Results are summarized in Fig. S- 3.


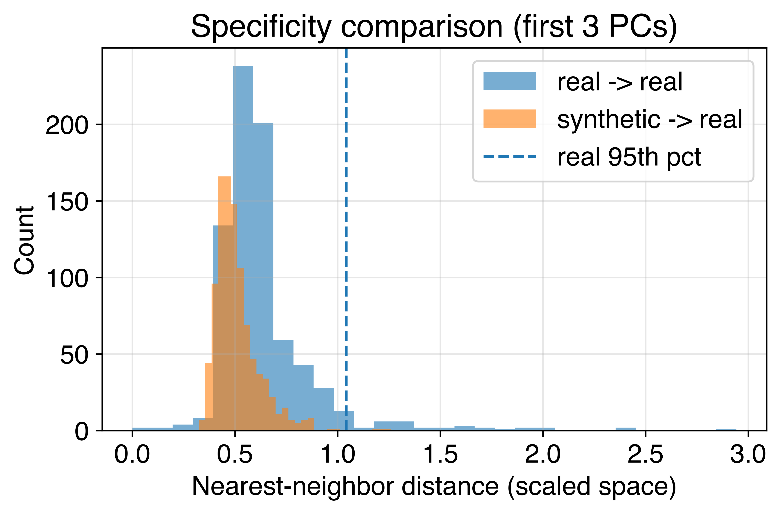


Fig. S- 3 Specificity of the 3-PC SSM. Nearest-neighbor distances in the standardized shape space. Blue: distances among real shapes (real/real). Orange: distances from 100 synthetic shapes – sampled from PCs 1–3 with scores truncated to ±2 SD – to their nearest real neighbor (synthetic/real). Dashed line = 95th percentile of the real/real distribution.

**Generalization (does the model reconstruct unseen shapes well?)**

Ten-fold cross-validation. Split the dataset into 10 folds. For each split:

- Fit on train (90%) to obtain $\mu$ and $V_{k}$ and project a held-out test shape $x$: the estimated scores

$$\hat{s}=V_{k}^{T}(x-\mu)$$

are the coordinates of $x$ in the retained subspace.

- The reconstruction

$$\hat{x}=\mu+V_{k}s$$

is the best rank-$k$ approximation of $x$ in that subspace.

Scaled in-plane mean squared error (MSE) (unitless) in standardized space is defined as

$$MSE_{scaled}=\frac{1}{2P}\left\| x-\hat{x} \right\|_{2}^{2},$$

where 2*P* counts the two in-plane coordinates (x,y) for each of the P correspondences; the axial coordinate z is standardized to fixed levels across shapes and is not included in the error.

Let $x_{mm}$ and $\hat{x}_{mm}$ denote the test and reconstructed shapes in millimeters using that specimen’s inverse scale factor; then

$$RMS_{mm}=\sqrt{\frac{1}{2P}\left\| x_{mm}-\hat{x}_{mm} \right\|_{2}^{2}} .$$

Similar train vs. test error distributions (both scaled MSE and RMS mm) indicate that the retained PCs reconstruct unseen anatomy as well as training shapes – i.e., no overfitting. Cross-validated distributions are shown in Fig. S- 4.


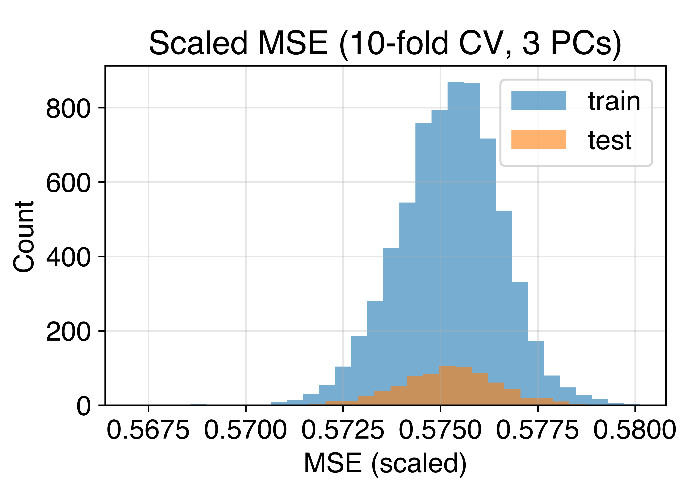


Fig. S- 4 Generalization under 10-fold cross-validation (3 PCs). Distributions of reconstruction error for training (blue) and held-out test (orange) shapes as scaled MSE (unitless). The near-identical train-test distributions indicate good generalization.

**Results of sex-specific SSMs**


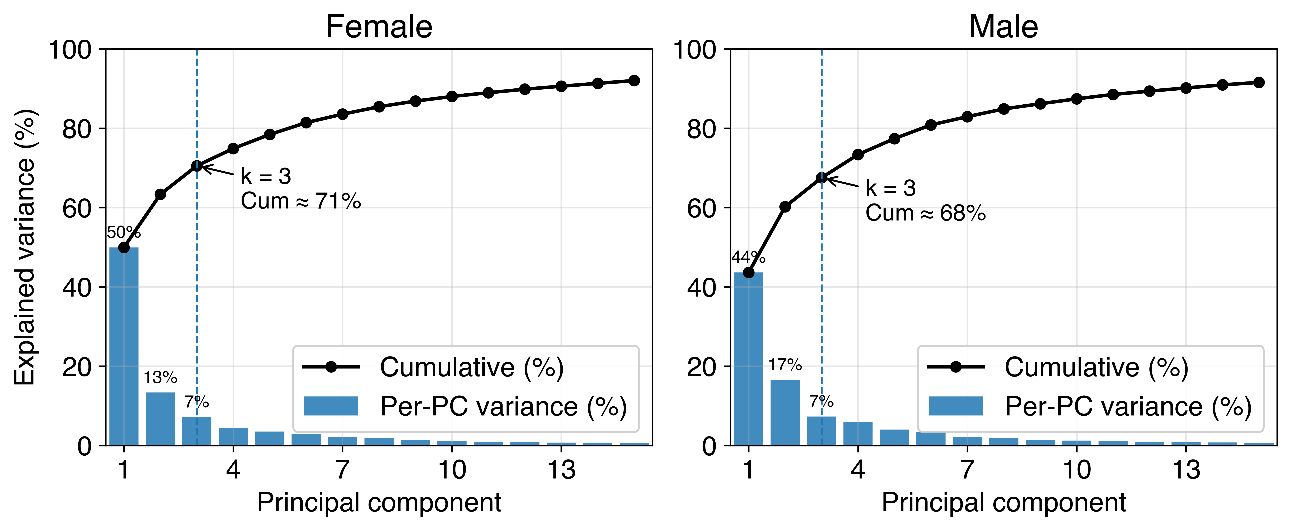


Fig. S- 5 Scree plots showing per-component explained variance (bars) and cumulative explained variance (line) for the first 15 PCs of the male and female SSM; the scree/elbow selection at PC = 3 is indicated (cumulative ≈ 68%).

**Linear regressions of shape coefficients and geometric properties**


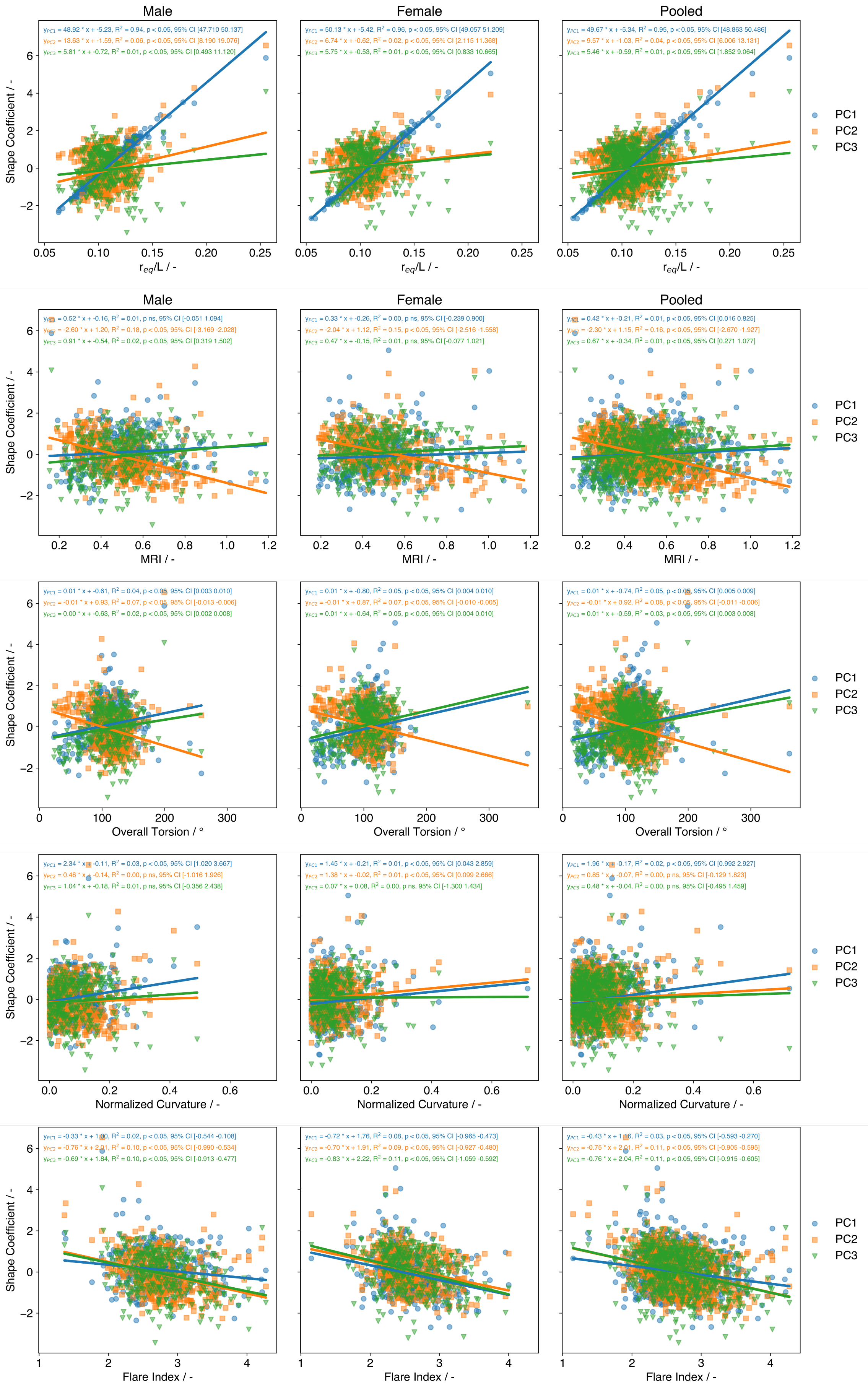


Fig. S-6 Linear regression of shape coefficients provided by the SSM and several geometrical characteristics.

**Score plots of first three principal components of pooled dataset**

**
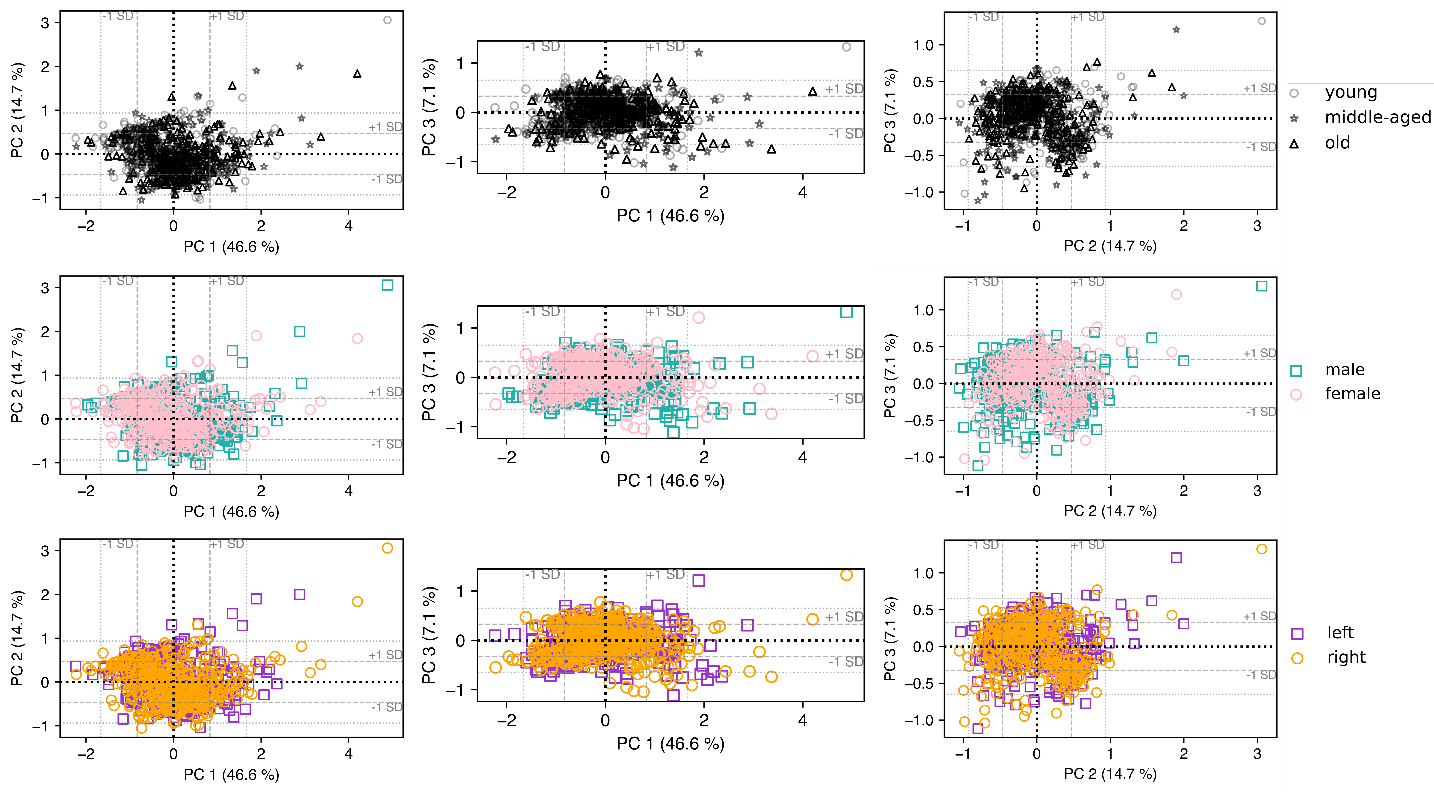
**

Fig. S-7 Score plots comparing PC1, PC2, and PC3 distinguishing age groups (top row), sex (middle row), and anatomical site (bottom row).
